# Major depressive episode in hospital workers during the Covid-19 pandemic in Brazil

**DOI:** 10.11606/s1518-8787.2022056004668

**Published:** 2022-11-18

**Authors:** Betina Daniele Flesch, Ana Laura Sica Cruzeiro, Maitê Peres de Carvalho, Laura Moreira Goularte, Felipe Mendes Delpino, Anaclaudia Gastal Fassa

**Affiliations:** I Universidade Federal de Pelotas Departamento de Medicina Social Programa de Pós-graduação em Epidemiologia Pelotas RS Brasil Universidade Federal de Pelotas. Departamento de Medicina Social. Programa de Pós-graduação em Epidemiologia. Pelotas, RS, Brasil; II Universidade Federal de Pelotas Faculdade de Medicina Pelotas RS Brasil Universidade Federal de Pelotas. Faculdade de Medicina. Curso de Psicologia. Pelotas, RS, Brasil; III Universidade Federal de Pelotas Faculdade de Medicina Pelotas RS Brasil Universidade Federal de Pelotas. Faculdade de Medicina. Curso de Terapia Ocupacional. Pelotas, RS, Brasil; IV Universidade Federal de Pelotas Faculdade de Enfermagem Programa de Pós-graduação em Enfermagem Pelotas RS Brasil Universidade Federal de Pelotas. Faculdade de Enfermagem. Programa de Pós-graduação em Enfermagem. Pelotas, RS, Brasil

**Keywords:** Patient Care Team, Depressive Disorder, Major, epidemiology, COVID-19, Working Conditions, Occupational Health

## Abstract

**OBJECTIVES:**

To estimate prevalence and factors associated with major depressive episode (MDE), emphasizing occupational aspects, in workers of a public teaching hospital that is a reference for Covid-19 treatment.

**METHODS:**

A cross-sectional study was carried out between October and December 2020, after the first peak of the pandemic, interviewing 1,155 workers. The prevalence of MDE was estimated using the Patient Health Questionnaire (PHQ-9) algorithm. Multivariate hierarchical analysis was conducted using Poisson regression to assess associated factors.

**RESULTS:**

MDE prevalence was 15.3% (95%CI: 13.3–17.5) and was higher among young, white and female workers, those with a family history of depression, resident professionals, nursing professionals, workers who were exposed to three or more situations of moral dilemma, and those who had to put off a physiological need until later. Having a risk factor for Covid-19, being a smoker and being physically inactive were also positively associated with MDE.

**CONCLUSIONS:**

The study points to the considerable prevalence of MDE among tertiary health care workers; reviewing work processes is essential to reduce occupational stress and minimize the effects of the pandemic on mental health, preventing those problems from becoming chronic.

## INTRODUCTION

The health sector accounts for more than 4% of the formally employed population in the country, being very important to the Brazilian economy^1^. People who work in tertiary care include nursing professionals (nurses, nursing technicians, and nursing auxiliaries), physicians, administrative workers, support workers, and other professionals, either hired directly or outsourced. Studies before the pandemic already pointed that these workers face long working hours, a precarious work environment, lack of supplies and personnel, a lot of time spent doing bureaucratic tasks, moral harassment, low autonomy, and little participation in decision-making processes^2,3^. Studies conducted in Brazil before the pandemic, mostly with nurses and nursing technicians, already highlighted the impact of their work on mental health, finding depressive symptom prevalence rates of between 40% and 50%, varying according to the instruments used to assess symptoms and levels of severity of the disease^4^.

The Covid-19 pandemic led to intensified work in hospitals, as well as increased concerns about biosafety and important changes in work processes. Health professionals face ethical and moral dilemmas in their routine, as they are forced to make quick and difficult decisions to save or reduce damage to individual or collective health. During the pandemic, resource shortages and treatment controversies further exacerbated this problem^7^. The health emergency situation with longer working hours, lack of individual protection equipment, lack of training, fear of contamination, and of contaminating family members increased workers’ burnout^8,9^.

The first studies on workers’ depression during the pandemic were developed in China using the Patient Health Questionnaire-9 (PHQ-9). This instrument is based on DSM-5 criteria which characterizes major depressive episode (MDE) as the presence of depressed, empty, or irritable mood, accompanied by somatic and cognitive changes that affect the individual’s functional capacity for at least one week^10^. The Chinese studies found depression prevalence rates of between 30% and 40%. The study by Lai et al.^11^, conducted with 1,257 health care workers who were caring for Covid-19 patients, found that 50.4% of the participants reported symptoms of depression. The study conducted by Kang et al.^12^ with 944 physicians and nurses found that 34.4% had mild depression symptoms (PHQ-9: 5.4), 22.4% had moderate symptoms (PHQ-9: 9.0), and 6.2% had severe symptoms (mean PHQ-9: 15.1) immediately following the peak of the pandemic(12). There was also a study conducted in Mexico that screened 5,938 health workers using the PHQ-2 test and found a depression prevalence rate of 37.7%.

Brazilian studies found a prevalence of depressive symptoms varying from 25% among hospital nurses to 60% among professionals from an intensive care unit^13,14^. Several studies are small, sampling less than 100 professionals, and the larger online-conducted studies often failed to reach a representative sample^14,15^.

We found the following online-conducted studies: one with 1,054 graduated health professionals (34.5% physicians, 19.1% nursing technicians, 14.2% nurses and 11.9% psychologists) that observed PHQ-9 scores suggestive of clinically significant depression, with higher rates among nursing technicians (68.2% with BP ≥ 50 and 68.7% with PHQ-9 ≥ 9) and front-line PS (61.3% with PB ≥ 50 and 58% with PHQ-9 ≥ 9)^16^. An online survey among 1,609 healthcare workers observed a depression prevalence of 57%, with the lowest scores among physicians having 38%^17^.

Depression in health professionals working during the pandemic was more frequent among women, nurses, and other health workers, in comparison to physicians, frontline health workers, and younger and less experienced health workers. Other factors positively associated with depression were bereavement, having a colleague infected with Covid-19 and/or quarantined, lack of emotional support, personal history of mental health problems, lack of adequate personal protective equipment, and exposure to moral dilemmas at work^11,12,18^.^.^ Few Brazilian studies evaluated factors associated with depression, showing a direct association with age, length of service in the profession and a positive association with job and work shift satisfaction^21,22^.

Considering that health care workers in Brazil already faced precarious working conditions before the Covid-19 pandemic and that resulted in a greater workload, this study seeks to estimate prevalence of MDE among tertiary health care workers facing Covid-19 in the municipality of Pelotas - RS and associated sociodemographic, behavioral, and occupational factors. The study will count with a large representative sample of hospital workers, allowing more knowledge about the impact of the pandemic on those workers’ mental health.

## METHODS

A cross-sectional study was conducted with all 1,731 workers at a Brazilian National Health System (SUS) teaching hospital to which patients were referred for Covid-19 treatment between October and December 2020, after the first peak of the pandemic. During the study, the weekly average was of 87 Covid-19 cases per 100,000 people in October, and of 64 cases per 100,000 people in November. The study was completed before the increase in cases by the delta variant occurred in December. The study was conducted in a medium-sized city in Southern Brazil and included workers hired by the Brazilian Hospital Services Company (EBSERH) under the Single Legal Regime (RJU) and outsourced workers who worked on-site or remotely during the Covid-19 pandemic.

The sample size to estimate the prevalence of MDE took into account an expected depression prevalence of 30%^12^, 95% confidence intervals (acceptable error of 2pp). Adding 10% for losses, a sample of 1,026 workers was estimated. To evaluate the associations, a confidence level of 95%, a statistical power of 80%, and exposed/non-exposed ratio of 1:8 (profession – variable that needs larger sample size), with a prevalence in non-exposed of 10% were considered. Adding 10% for losses and 15% to control for confounding factors, a sample of 958 workers would be needed to estimate risks above 2.

The study investigated sociodemographic aspects of the health workers, including age, skin color (categories used by the Brazilian Institute of Geography and Statistics - IBGE), biological sex, level of education, socioeconomic level according to Brazil Social Classification Criteria (CCEB 2020) by Brazilian Association of Research Companies (ABEP) as well as family history of depression. Regarding occupational aspects, profession was categorized in nurses, physicians, residents, nursing auxiliaries and technicians, support workers, administrative workers, and other health workers (nutritionists, physiotherapists, among others). The residents’ category included workers of different professions who were doing training in the hospital. Among the occupational aspects, the type of contractual link with the teaching hospital was also evaluated, whether the health worker was working or had worked in sectors intended for Covid-19 patients, whether they had asked to change from one sector to another during the pandemic, and whether they had worked remotely any time during the pandemic.

Taking workloads into consideration, exposure to moral dilemma was examined by investigating whether workers witnessed clinical procedures they considered to be inappropriate, witnessed colleagues’ attitudes towards them or their patients they disagreed with, felt pressured by colleagues or superiors to act in ways they disagreed with regarding clinical procedures and contractual norms, and felt pressured by patients or family members to act in ways they disagreed with. The questions were subdivided in never or hardly ever and occasionally/frequently, then a score ranging from one to five was derived from the sum of the occasionally/frequently answers to these questions.

The study also assessed whether workers had to put off any physiological need until later they had during the work shift, such as drinking water, eating, and going to the bathroom; whether the number of health workers available was sufficient, whether they had adequate physical space to perform their activities and whether they had a place to rest.

Occupational stress was measured by the Portuguese version of the Job Stress Scale (JSS) validated in Brazil^23^, which includes the demand, control, and social support dimensions (internal consistency estimates (Cronbach’s alpha) were, respectively, 0.79 demand, 0.67 control, and 0.85 social support), classifying work as a high or low strain, active or passive job, as well as the presence or absence of social support. This scale is an abridged version of the Job Content Questionnaire (JCQ)^24^. Regarding behavioral aspects, we determined tobacco and alcoholic beverage consumption, as well as practicing of physical activity.

The MDE was investigated using the Portuguese version of the Patient Health Questionnaire-9 (PHQ-9)^25^ and validated for Brazil. This instrument has nine questions that assess how each of the symptoms of MDE presents, as described in the Diagnostic and Statistical Manual of Mental Disorders (DSM-IV). MDE was investigated using the PHQ-9 algorithm (sensitivity 42.5%; 95%CI: 27.0–59.1 and specificity 95.3%; 95%CI: 92.8–97.2)^25^, considering it to exist when five or more symptoms were present, provided that at least one was depressed mood or anhedonia and that each symptom had occurred for “a week or more” or “almost every day”, except for symptom nine, when the criteria used were occurrence for “less than a week”, “a week or more”, or “almost every day”. The prevalence of depression was also evaluated, according to severity levels, classified as: without depression (1 to 4 points), mild depression (5 to 9), moderate depression (10 to 14), moderately severe depression (15 to 19), or severe depression (20 to 27 points)^26^.

The workers were invited to participate in the study through their institutional e-mail, posters, and the hospital’s website and social media. The researchers also contacted hospital sector managers to identify and release workers to participate in the research. At the end of the fieldwork, those who had still not answered the questionnaire were contacted by telephone.

The study was conducted using a self-administered digital questionnaire on tablets answered at the workplace or online outside the workplace. The study had four supervisors and 15 trained interviewers who were present at the data collection site and accessible by telephone, to welcome workers, guide them in completing the questionnaire and answer queries. All study personnel received training and personal protective equipment, following all biosafety protocols.

The data were analyzed using the STATA 15.1 program. A descriptive analysis of the independent variables and the outcome was performed in order to characterize the sample. Association between the independent variables and the outcome was estimated using prevalence ratios and their respective 95% confidence intervals (95%CI), the chi-square test for heterogeneity and the linear trend test. Multivariate hierarchical analysis was performed using Poisson^27^ regression with robust variance and backward selection. To adjust for confounding factors, variables associated with the outcome with a p-value < 0.20 were kept in the model. Variables with a p-value < 0.05 were considered to be associated.

The study project was approved by the Research Ethics Committee of the Faculty of Medicine of the *Universidade Federal de Pelotas* as per Opinion No. 4.040.039 issued on May 21, 2020. All participants were informed about the research topic, the protection of identity regarding the information provided, and the right to not participate or to stop participating at any time. Those who agreed to participate in the research signed an informed consent form.

## RESULTS

A total of 1,155 workers from different sectors of the hospital were interviewed, with a response rate of 66.7%. In the population studied, 39.9% were between 30 and 39 years old; 74.4% self-reported having white skin color; 76% were female; 38.5% had concluded postgraduate courses; 53.3% belonged to economic level B and 50.8% reported a family history of depression (Table 1).

**Table 1:** Description of the sample according to sociodemographic and occupational characteristics of tertiary health care workers during the Covid-19 pandemic. Pelotas, RS, Brazil, 2021 (n = 1,155).

Variables	n (%)	MDE (%)	p
**Sociodemographic aspects**			
Age			
19–29	133 (11.6)	19.6	0.043
30–39	458 (39.9)	15.7	
40–49	369 (32.1)	16.5	
≥ 50	189 (16.4)	9.7	
Skin color or race			
White	859 (74.4)	16.1	0.022
Black	141 (12.2)	18.4	
Brown	143 (12.4)	7.7	
Yellow	7 (0.6)	0	
Indigenous	5 (0.4)	40.0	
Sex			
Male	277 (24.0)	8.3	< 0.001
Female	878 (76.0)	17.5	
Schooling			
Illiterate/technical course	329 (28.9)	13.4	0.017
Complete/incomplete higher education	371 (32.6)	19.7	
Complete postgraduate course	437 (38.5)	13.1	
Socioeconomic level (ABEP)			
A	163 (14.4)	11.7	0.358
B	605 (53.3)	15.7	
C-D-E	367 (32.3)	16.4	
Family history of depression			
No	568 (49.2)	31.1	< 0.001
Yes	587 (50.8)	68.9	
**Occupational aspects**			
Profession			
Nurses	136 (11.8)	18.5	0.009
Physicians	107 (9.3)	7.5	
Residents	54 (4.7)	29.6	
Nursing auxiliaries and technicians	316 (27.4)	16.5	
Support	242 (20.9)	15.0	
Administrative	111 (10.1)	15.7	
Other health workers	183 (15.8)	11.7	
Contractual link			
Employed directly	718 (62.2)	15.2	0.019
Resident	54 (4.7)	29.6	
Emergency/temporary	112 (9.7)	11.6	
Outsourced	271 (23.4)	14.4	
**Work organization**			
Worked in Covid-19 ICU			
No	989 (85.6)	16.2	0.049
Yes	166 (14.4)	10.2	
Worked in Covid-19 ward			
No	987 (85.5)	16.0	0.118
Yes	168 (14.5)	11.3	
Asked to change sectors during the pandemic			
No	1,061 (91.9)	14.1	< 0.001
Yes	94 (8.1)	29.8	
Worked remotely			
No	916 (79.3)	15.0	0.496
Yes	239 (20.7)	16.7	

N (%): number of observations (percentage); MDE (%): prevalence of major depressive episode according PHQ-9 algorithm. ABEP: Brazilian Association of Research Companies; CCEB 2020: Brazil Social Classification Criteria.

p-value - Person Chi^2^ test.

Missing data: age (6), schooling (18), socioeconomic level (20).

Most of the surveyed health workers were nursing technicians and nursing auxiliaries (27.4%), followed by support workers (security, hygiene, maintenance, etc.) (20.9%); as for their contractual link with the teaching hospital, 62.2% of the workers were permanent, 23.4% were outsourced, 9.7% had emergency/temporary contracts, and 4.7% were resident professionals. As for the work routine, 14.4% worked in the Covid-19 ICU; 14.5% worked in Covid-19 wards; 8.1% asked to change sectors in the hospital during the pandemic and 20.7% worked remotely at some time during the pandemic (Table 1).

Of the workers interviewed, 37.7% reported having witnessed during the pandemic situations related to clinical procedures they judged incorrect, and 33.7% witnessed attitudes among colleagues or towards patients they disagreed with; 15.2% felt pressured by colleagues or superiors to act in ways they disagreed with in relation to clinical procedures; 11.3% felt pressured by colleagues or superiors to act in disagreement with contractual norms and 10.8% felt pressured by patients or family members to act in ways they disagreed with. Among the workers, 15.4% experienced three or more situations related to moral dilemma (Table 2).

**Table 2:** Description of moral dilemma among tertiary health care workers during the Covid-19 pandemic. Pelotas, RS, Brazil, 2021 (n = 1,155).

Variables	n (%)	% MDE	p
**Moral dilemma**			
Witnessed clinical procedures in disagreement with what he/she considers correct			
Never or hardly ever/rarely	719 (62.3)	11.8	< 0.001
Sometimes	348 (30.1)	17.5	
Frequently	88 (7.6)	35.2	
Witnessed attitudes of colleagues or attitudes towards patients in disagreement with what he/she considers correct			
Never or hardly ever/rarely	766 (66.3)	11.8	< 0.001
Sometimes	318 (27.5)	20.1	
Frequently	71 (6.2)	32.4	
Felt pressured by colleagues or superiors to act in disagreement with what he/she considers correct in relation to clinical procedures			
Never or hardly ever/rarely	979 (84.8)	13.0	< 0.001
Sometimes	141 (12.2)	24.1	
Frequently	35 (3)	45.7	
Felt pressured by colleagues or superiors to act contrary to contractual norms			
Never or hardly ever/rarely	1,025 (88.7)	13.5	< 0.001
Sometimes	106 (9.2)	28.3	
Frequently	24 (2.1)	37.5	
Felt pressured by patients or their family members to act in disagreement with what he/she considers correct			
Never or hardly ever/rarely	1,030 (89.2)	13.3	< 0.001
Sometimes	99 (8.6)	30.3	
Frequently	26 (2.2)	38.5	
Moral dilemma score			
0	589 (51.0)	10.2	< 0.001
1	196 (17.0)	14.8	
2	192 (16.6)	16.7	
≥ 3	178 (15.4)	31.5	

N (%): number of observations (percentage); MDE (%): prevalence of major depressive episode according PHQ-9 algorithm.

p-value - Person Chi^2^ test.

Around half of the workers had to put off physiological needs until later, such as eating, drinking water, and going to the bathroom; 38.1% of the interviewees considered the hospital lacked enough workers; 42.4% considered that the hospital lacked enough physical space to perform the work and 48.8% informed there was no place for them rest (Table 3).

**Table 3:** Description of workloads among tertiary health care workers during the Covid-19 pandemic. Pelotas, RS, Brazil, 2021 (n = 1,155).

Variables	n (%)	MDE (%)	p
**Workloads**			
Needed to put off physiological needs until later, such as:			
Drinking water			
No	606 (52.5)	12.7	0.009
Yes	549 (47.5)	18.2	
Going to the bathroom			
No	623 (53.9)	12.4	0.002
Yes	532 (46.1)	18.8	
Eating			
No	582 (50.4)	13.0	0.020
Yes	573 (49.6)	17.8	
Needed to put off any physiological need until later			
No	504 (43.6)	11.3	0.001
Yes	651 (56.4)	18.4	
Sufficient numbers of staff			
No	440 (38.1)	19.3	0.003
Yes	715 (61.9)	12.9	
Adequate physical space			
No	490 (42.4)	20.8	< 0.001
Yes	665 (57.6)	11.3	
Adequate place for resting during breaks			
No	446 (48.8)	21.5	< 0.001
Yes	468 (51.2)	8.8	
**Occupational stress: demand, control, and social support – Job stress scale (JSS)**			
High job strain			
No	922 (79.8)	13.2	< 0.001
Yes	233 (20.2)	23.6	
Active job			
No	768 (66.5)	14.7	0.417
Yes	387 (33.5)	16.5	
Passive job			
No	865 (74.9)	16.3	0.112
Yes	290 (25.1)	12.4	
Low job strain			
No	910 (78.8)	17.1	0.002
Yes	245 (21.2)	8.9	
Social support			
Low	408 (35.3)	23.5	< 0.001
High	747 (64.7)	10.8	

N (%): number of observations (percentage); MDE (%): prevalence of major depressive episode according PHQ-9 algorithm.

p-value - Person Chi^2^ test.

Missing data: adequate place for resting during breaks (239).

According to the JSS occupational stress scale, 20.2% were exposed to high job strain, 33.5% to active job, 25.1% to passive work and 21.2% to low job strain and 35.3% had low social support (Table 3).

Among the workers, 42.5% had one or more Covid-19 risk factors, 74.2% were nonsmokers, and 52.4% declared themselves physically active. Regarding mental health, 16.2% of the workers consulted with a psychiatrist or psychologist and 20.2% used medication for mental health. MDE prevalence was 15.3% (95%CI: 13.3–17.5) and the evaluation of the depression severity levels pointed that 15.5% had moderate depression, 8.6% moderate severe depression, and 4.4% severe depression (Table 4).

**Table 4:** Description of the sample according to behavioral aspects, Covid-19 risk factors and prevalence of major depressive episode among tertiary health care workers during the Covid-19 pandemic. Pelotas, RS, Brazil, 2021 (n = 1,155).

Variables	n (%)	MDE (%)	p
Behavioral aspects and Covid-19 risk factors			
Tobacco smoking			
Non-smoker	857 (74.2)	13.4	< 0.001
Former smoker	182 (15.8)	14.8	
Smoker	116 (10.0)	30.2	
Physical activity			
Inactive	550 (47.6)	21.8	< 0.001
Active	605 (52.4)	9.4	
Covid-19 risk factors			
No	664 (57.5)	10.4	< 0.001
Yes	491 (42.5)	22.0	
**Mental health**			
Consultation with psychologist or psychiatrist			
No	967 (83.8)	13.3	< 0.001
Yes	187 (16.2)	25.7	
Use of mental health medication			
No	914 (79.8)	10.7	< 0.001
Yes	231 (20.2)	33.7	
**Patient health questionnaire-9 (PHQ-9)**			
	n (%)	95%CI	
Major depressive episode			
No	978 (84.7)	82.5–86.6	
Yes	177 (15.3)	13.3–17.5	
Depression severity levels			
Without depression (< 5)	481 (41,7)	38,8–44,5	
Mild (5/9)	345 (29,9)	27,3–32,6	
Moderate (10/14)	179 (15,5)	13,5–17,7	
Moderate severe (15/19)	99 (8,6)	7,1–10,3	
Severe (20/27)	51 (4,4)	3,4–5,7	

N (%): number of observations (percentage); 95%CI: 95% confidence interval; MDE (%): prevalence of major depressive episode according PHQ-9 algorithm.

Depression severity levels - according PHQ-9 cut-off points.

p-value - Person Chi^2^ test.

Missing data: consultation with psychologist or psychiatrist (1).

In the adjusted analysis, prevalence of MDE was 50% lower in workers aged 50 years or older (PR = 0.47; 95%CI: 0.27–0.82) when compared with those aged 19–29 years and in those of brown skin color when compared with those of white/yellow (Asian) skin color. MDE was twice as high among female workers and those with a family history of depression (PR = 1.93; 95%CI: 1.28–2.91; PR = 2.05; 95%CI: 1.52–2.77, respectively). When compared to physicians, the prevalence ratio of MDE among resident professionals was 3.83 (95%CI: 1.69–8.70), followed by nursing technicians and nursing auxiliaries with 2.25 (95%CI: 1.10–4.57), and nurses with 2.20 (95%CI: 1.02–4.77) (Table 5).

**Table 5:** Factors associated with major depressive episode among tertiary health care workers during the Covid-19 pandemic. Pelotas, RS, Brazil, 2021 (n = 1,155).

Variables	PR (95%CI)	p	PR (95%CI)	p
	
	Crude analysis		Adjusted analysis	
**1**^st^ **level: sociodemographic variables**				
Age		0.057		0.054
19–29	Ref		Ref	
30–39	0.80 (0.54–1.21)		0.81 (0.54–1.20)	
40–49	0.85 (0.56–1.28)		0.87 (0.58–1.30)	
≥ 50	0.46 (0.26–0.81)		0.47 (0.27–0.82)	
Skin color or race		0.024		0.014
White/yellow	Ref		Ref	
Black/indigenous	1.20 (0.83–1.74)		1.33 (0.93–1.90)	
Brown	0.48 (0.27–0.87)		0.51 (0.28–0.92)	
Sex		< 0.001		0.002
Male	Ref		Ref	
Female	2.15 (1.60–2.89)		1.93 (1.28–2.91)	
Family history of depression		< 0.001		< 0.001
No	Ref		Ref	
Yes	2.15 (1.62–2.95)		2.05 (1.52–2.77)	
**2**^nd^ **level: work organization**				
Profession		0.008		0.038
Physicians	Ref		Ref	
Nurses	2.46 (1.16–5.23)		2.20 (1.02–4.77)	
Residents	3.96 (1.81–8.67)		3.83 (1.69–8.70)	
Nursing auxiliaries and technicians	2.24 (1.10–4.57)		2.25 (1.10–4.57)	
Support	1.99 (0.96–4.14)		2.07 (0.99–4.32)	
Administrative	2.06 (0.93–4.54)		2.13 (0.97–4.71)	
Other health workers	1.53 (0.70–3.34)		1.61 (0.74–3.53)	
Works in the Covid-19 ICU		0.058		0.090
No	Ref		Ref	
Yes	0.63 (0.39–1.02)		0.66 (0.40–1.07)	
Asked to change sectors during the pandemic		< 0.001		< 0.001
No	Ref		Ref	
Yes	2.12 (1.50–2.99)		1.89 (1.32–2.71)	
**3**^rd^ **level: workloads**				
Moral dilemma score		< 0.001		0.001^a^
0	Ref		Ref	
1	1.45 (0.96–2.20)		1.33 (0.82–2.15)	
2	1.64 (1.10–2.43)		1.37 (0.86–2.16)	
≥ 3	3.09 (2.23–4.27)		2.06 (1.36–3.13)	
Needed to put off any physiological need until later		0.001		0.048
No	Ref		Ref	
Yes	1.63 (1.22–2.19)		1.42 (1.01–2.01)	
Adequate place for resting during breaks		< 0.001		0.002
No	Ref		Ref	
Yes	0.41 (0.29–0.57)		0.57 (0.40–0.81)	
Low job strain		0.003		0.023
No	Ref		Ref	
Yes	0.53 (0.35–0.81)		0.55 (0.32–0.92)	
Social support		< 0.001		0.004
Low	Ref		Ref	
High	0.46 (0.35–0.60)		0.60 (0.43–0.85)	
**4**^th^ **level: behavioral variables and comorbidities**				
Covid-19 risk factors		< 0.001		< 0.001
No	Ref		Ref	
Yes	2.12 (1.60–2.80)		1.85 (1.37–2.50)	
Tobacco smoking		< 0.001		0.001
Non-smoker	Ref		Ref	
Smoker	2.21 (1.61–3.03)		1.91 (1.31–2.79)	
Physical activity		< 0.001		< 0.001
Inactive	Ref		Ref	
Active	0.43 (0.32–0.58)		0.53 (0.39–0.73)	

PR: prevalence ratio; 95%CI: 95% confidence interval; p-value: heterogeneity test.

^a^ p-value for linear trend.

Professionals who worked in the Covid-19 ICU had around 30% lower prevalence of MDE compared to other professionals (PR = 0.66; 95%CI: 0.40–1.07). MDE was two times higher among professionals who asked to change sectors during the pandemic (PR = 1.89; 95%CI: 1.32–2.71) or who were exposed to three or more moral dilemma situations during their professional activity in the pandemic (PR = 2.06; 95%CI: 1.36–3.13). Professionals who had to put off physiological needs until later had 42% higher prevalence of MDE (PR = 1.42; 95%CI: 1.01–2.01). Having an adequate place to rest during breaks was a protective factor for depression (PR = 0.57; 95%CI: 0.40–0.81) (Table 5).

Low job strain (PR = 0.55; 95%CI: 0.32–0.92) and availability of social support (PR = 0.60; 95%CI: 0.43–0.85) were associated with a 50% reduction in MDE. Professionals with Covid-19 risk factors had 85% higher prevalence of depression (95%CI: 1.37–2.50); workers who smoked had 1.91 times (95%CI: 1.31–2.79) higher prevalence of depression and physically active workers had 53% less depression compared with physically inactive workers (95%CI: 0.39–0.73) (Table 5).

## DISCUSSION

Hospital workers presented high prevalence of MDE and of moderate to severe depression, a condition that causes great limitations, affecting not only the ability to work, but also personal life. The use of mental health medication is also high, however the percentage of workers that consulted with psychologists or psychiatrists is low, explaining its high use. Studies highlight the importance of these workers’ psychological monitoring, especially after the health emergency. It also emphasizes the need of self-medication awareness, considering the easy access of health professionals to medicines^5,28^.

The study indicates important associations between some occupational aspects and MDE, as the higher prevalence of MDE among resident professionals, nursing technicians and auxiliaries, and nurses when compared to physicians, as well as, among workers who requested a change of sector in the teaching hospital during the pandemic, were exposed to three or more situations of moral dilemma or needed to put off a physiological need until later. Workers who had been working in the Covid-19 ICU, had an adequate place to rest, were exposed to low-demand work, and had social support reported less MDE.

Studies in other countries during the pandemic found MDE prevalence of around 10% in physicians and 20% in nurses^19,29^, these being similar to the prevalence rates found in this study. Nurses and nursing technicians and auxiliaries are the most numerous group of workers in hospitals, being responsible for tasks related to direct care of patients, such as those related to hygiene and comfort, as well as bureaucratic tasks^2,3^. Besides their formal employment, these workers, mostly women, also care for children and older adults, as well as doing household chores, demands that can overburden them physically, mentally and emotionally^19^.

The literature pointed a prevalence of MDE in young trainee workers, accounting for 20% of workers^19,29,30^, which is lower than the prevalence found in resident professionals in this study. Being a young professional or a resident was associated with higher prevalence of depression and other mental health problems among health care workers during the response to Covid-19^12,29^. Studies observed that the insecurity and concern about the future inherent to the academic period of life, added to overburdening, emotional exhaustion and lack of professional experience in dealing with the demands of the pandemic can lead to the exacerbation of depressive symptoms among resident professionals^31,32^. The studies did not address other categories of hospital workers, as support and administrative workers, that also presented high prevalence of MDE^11,12^.

Providing direct care to Covid-19 patients has been associated with higher levels of depressive symptoms, anxiety and post-traumatic stress disorder^33,34^. In the present study the negative association between working in the Covid-19 ICU and MDE may be related to the healthy worker effect, since only the healthiest professionals able to work under great pressure continue working in ICUs, especially during a health emergency.

Moral dilemma is characterized as a conflict between doing the ethical versus the possible at a given moment, such as a decision between personal life and professional life, or between justice and compassion^35^. In the period of the fight against Covid-19, health workers were exposed to moral dilemmas related to the scarcity of equipment and supplies, such as ICU beds, respirators, anesthetics, which determines life and death. In addition, the dissemination of anti-scientific information led health workers to be pressured by government officials and health service users to use treatment lacking scientific evidence of effectiveness against Covid-19^36^. In agreement with the literature, our study found that being exposed to situations of moral dilemma at work, such as witnessing or being pressured to act against one’s principles, is associated with a greater occurrence of depression among healthcare workers^20^.

Work overload reduces break time and, along with the flexibilization of labor laws, causes an increase in working hours. Scarcity of PPEs makes health workers avoid leaving the sector where they work to avoid having to change them. These aspects, along with a lack of appropriate places to rest, lead health workers to put off physiological needs until later, causing physical and emotional exhaustion^37^

Similar to this study, several articles point to social support as a protective factor for depression among health care workers during the Covid-19 pandemic^38^. Health workers recognize the importance of social support, indicating that between 25% and 30% of them would like to receive emotional, psychological, and crisis management^38^. A study among nursing technicians before the pandemic found that having a high-demand job, according to the JSS, can even double the prevalence of depression. This is consistent with the negative association between low job strain and MDE^39^.

This study was consistent with the literature pointing that being younger, female, smoker, physically inactive, and reporting a family history of depression were associated with MDE^34,40,41^. The higher prevalence of depression among those of white skin color compared with those of brown skin color contradicts studies prior to the pandemic^42^ and could indicate that the brown-skinned population is selected, and might have higher resilience than those with white skin color in facing the health emergency^29^.

One study found that fear of infection was associated with depression during the pandemic^18^. Greater MDE prevalence among workers with Covid-19 risk factor agrees with the findings of Shacham et al.^43^, and may be related to the dilemma between the need to keep working and exposure at work, since by belonging to a risk group they have a greater chance of becoming severely ill.

This is one of the first Brazilian studies on the mental health of hospital workers in the context of Covid-19. The study has a large sample size, allowing to evaluate occupational factors associated with MDE. The desire to respond to what would be considered socially appropriate might generate a social desirability bias (information bias) underestimating MDE. This bias is reduced by using a self-administered survey, providing more adequate conditions to address mental health, and by using PHQ-9, a standardized instrument, validated for the Brazilian population. The PHQ-9 allows to determine MDE and depression severity levels, however, MDE is not estimated in other health care worker studies, limiting the similarity of the findings. The study has a good response rate, but non-respondents and the lack of those who were off work could result in selection bias, underestimating the outcome.

## CONCLUSION

MDE prevalence is high among hospital workers, especially nursing workers and residents, and several occupational aspects are contributing to this mental health problem. It is necessary to examine work processes in hospitals, promote more horizontal, participatory relationships and teamwork, avoid work intensification, and value workers. The health of hospital workers is a very important subject but, considering the sanitary emergency and its potential effects on workers’ health, the monitoring gained even more relevance. In this sense, it is important to develop not only cross-sectional studies, but also cohort studies allowing to examine the occurrence of chronic conditions such as post-traumatic stress and its associated factors. Hospital occupational health services need to perform health surveillance in order to identify the main problems arising from this health emergency period, triggering health promotion actions and psychological support to those who need it.
